# Supplementary material for: Tick-borne coinfections modulate CD8+ T cell response and progressive leishmaniosis
Source: Infect Immun. 2025 Jul 31;93(9):e00182-25. doi: 10.1128/iai.00182-25 (PMC12418745; doi:10.1128/iai.00182-25)
Supplement: Supplemental material — Fig. S1 to S6; Tables S1 and S2. [file iai.00182-25-s0001.pdf]

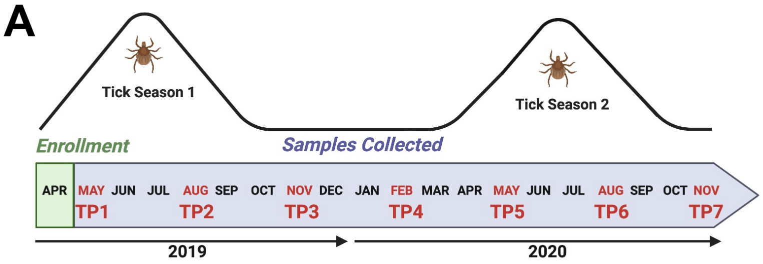


**B**

**Cohort age and sex distribution**

| | Site A (N=19) | Site B (N=17) | Site C (N=14) | Total (N=50) |
|---|---|---|---|---|
| **Age (%)** | | | | |
| 0-2 yrs | 9 (47.4%) | 9 (52.9%) | 3 (21.4%) | 21 (42%) |
| 3-5 yrs | 7 (36.8%) | 8 (47.1%) | 9 (64.3%) | 24 (48%) |
| ≥6 yrs | 3 (15.8%) | 0 (n/a) | 2 (14.3%) | 5 (10%) |
| **Sex (%)** | | | | |
| Male | 10 (52.6%) | 10 (58.8%) | 6 (42.9%) | 26 (52%) |
| Female | 9 (47.4%) | 7 (41.2%) | 8 (57.1%) | 24 (48%) |

**C**

**Cohort tick preventative treatment distribution by age and sex at enrollment**

| | Tick Preventative (N=25) | Placebo (N=25) | Total (N=50) |
|---|---|---|---|
| **Age (%)** | | | |
| 0-2 yrs | 11 (22%) | 10 (20%) | 21 (42%) |
| 3-5 yrs | 12 (24%) | 12 (24%) | 24 (48%) |
| ≥6 yrs | 2 (4%) | 3 (6%) | 5 (10%) |
| **Sex (%)** | | | |
| Male | 13 (26%) | 13 (26%) | 26 (52%) |
| Female | 12 (24%) | 12 (24%) | 24 (48%) |

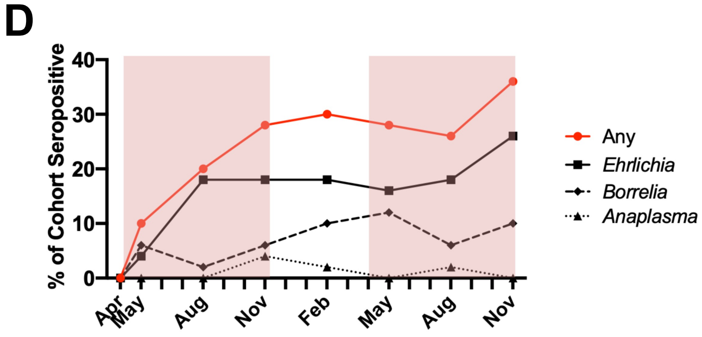


**E**

**Endpoint cohort TBC seroconversion status by treatment group**

| | TBC- (N=19) | TBC+ ≤ 6 Months (N=11) | TBC+ ≥ 12 Months (N=8) | Total (N=38) |
|---|---|---|---|---|
| **Tick Preventative** | 11 (58%) | 6 (55%) | 3 (38%) | 3 (53%) |
| **Placebo** | 8 (42%) | 5 (45%) | 5 (63%) | 5 (47%) |

**Supplemental Figure 1. Study timeline and cohort demographics.**
(A) Study timeline showing major collection time points in red occurring every three months over an 18-month period. Typical tick season overlaid. (B) Study cohort age and sex distribution at enrollment across the three study sites. (C) Study cohort age and sex distribution at enrollment by tick preventative treatment group. (D) Percent of cohort seropositive for one or more tick-borne pathogens listed by IDEXX 4Dx SNAP test at each major study time point. (E) Distribution of positive IDEXX 4Dx SNAP test duration results by tick preventative treatment group at the conclusion of the main study period. Includes only dogs included in the final analyses. TBC: tickborne coinfection seropositive.

| LeishVet Stage and Defining Features | | | Physical Clinical Signs | Clinicopathological Abnormalities |
|---|---|---|---|---|
| **Stage 0** | **Healthy** | **Seronegative. No clinical signs of disease. No clinicopathological findings.** | No clinical signs observed | None |
| **Stage 1** | **Mild Disease** | **Negative to low antibody levels. Mild clinical disease. No clinicopathological findings and normal renal profile.** | Lymphadenopathy (2 or more enlarged lymph nodes), dermatitis, conjunctivitis, | None |
| **Stage 2** | **Moderate Disease** | **Low to high antibody levels. Additional clinical signs. Clinicopathological findings of non-regenerative anemia, hyperglobulinemia, serum viscosity syndrome or hypoalbuminemia. Normal renal profile to mild proteinuria.** | Cutaneous lesions, anorexia, weight loss (Body Condition Score < 4), epistaxis, rough coat, or onychogryphosis. Splenomegaly. | Non-regenerative anemia (low RBCs with low or normal reticulocytes, or low hematocrit with low RBCs and low or normal reticulocytes), Hyperglobulinemia, or Hypoalbuminemia |
| **Stage 3** | **Severe Disease** | **Medium to high antibody levels. Additional clinical signs related to immune-complex lesions. Additional clinicopathological findings of chronic kidney disease: IRIS stage I and II.** | Uveitis, arthritis. | IRIS Stage 2: Creatinine 1.4-2.8 mg/dl or SDMA 18-35 ug/dl |
| **Stage 4** | **Extreme Severe Disease** | **Additional clinical signs such as pulmonary thromboembolism, or nephrotic syndrome. Additional clinicopathological findings of end stage renal disease: IRIS stage III and IV** | Deteriorating condition. | IRIS Stage 3: Creatinine 2.9-5 mg/dl or SDMA 36-54 ug/dl |

**Supplemental Table 1. Leishmaniosis clinical scoring system used to assign LeishVet stage to study dogs.** For each clinical stage, defining features as described in Solano-Gallego et al. are shown in bold. For the study dogs, physical signs and laboratory abnormalities used to assign a clinical stage are shown in the right two columns.

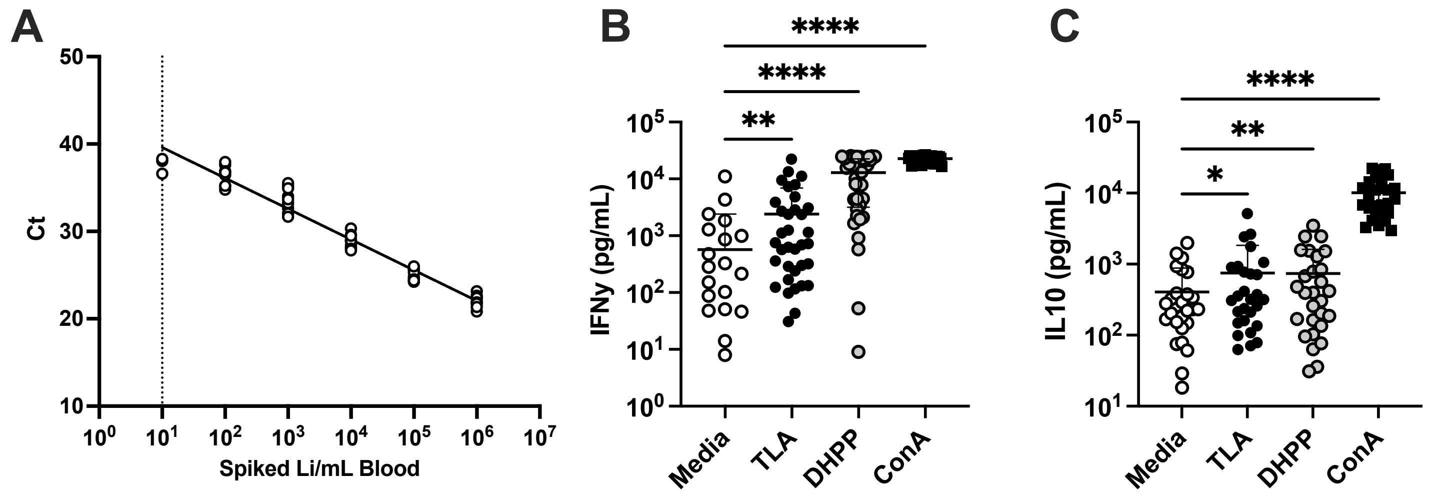


**Supplemental Figure 2.** Blood parasite burden RT-qPCR standard curve (A) and representative IFNy (B) and IL-10 (C) ELISA result.

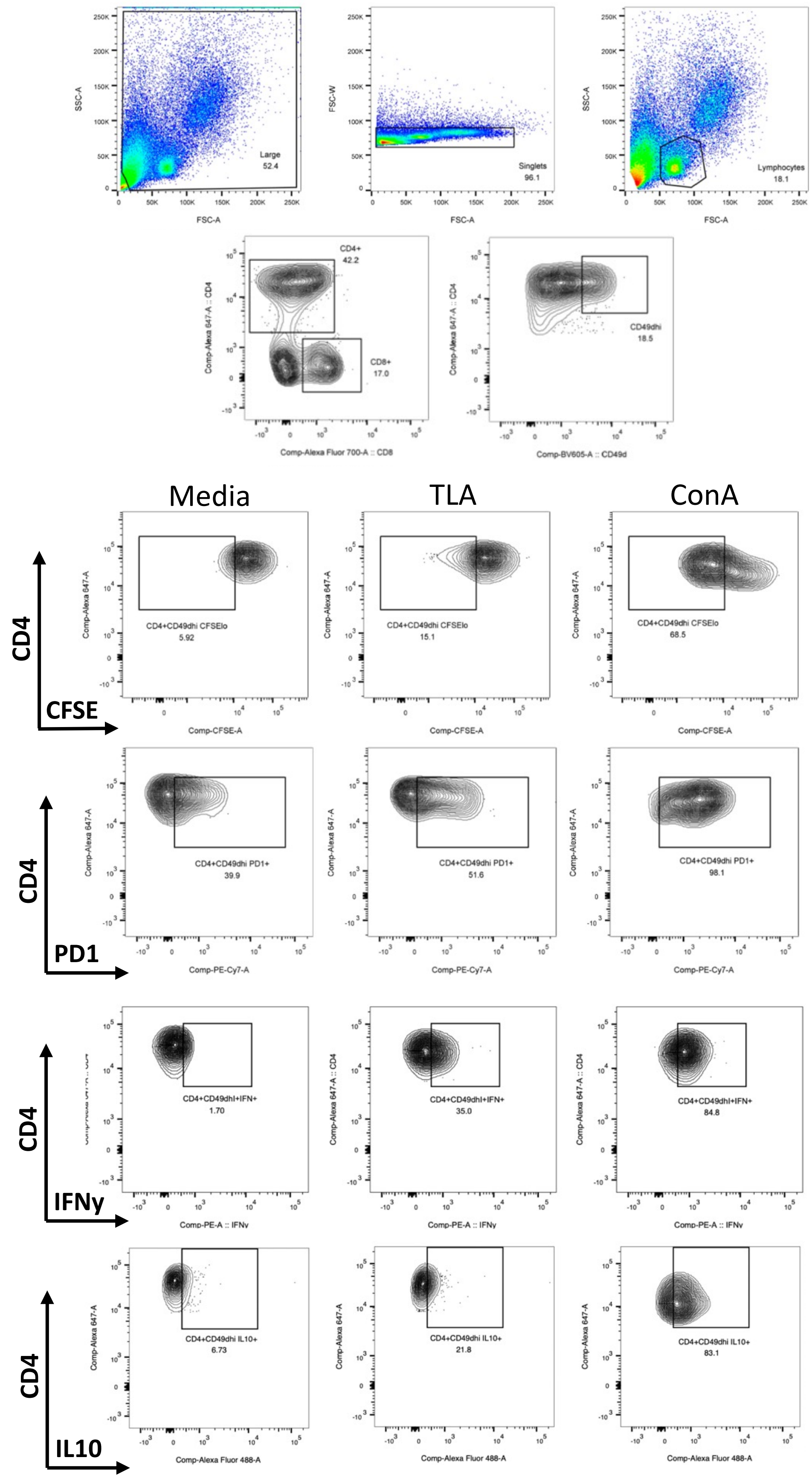


**Supplemental Figure 3.** Representative flow cytometry gating strategy example.

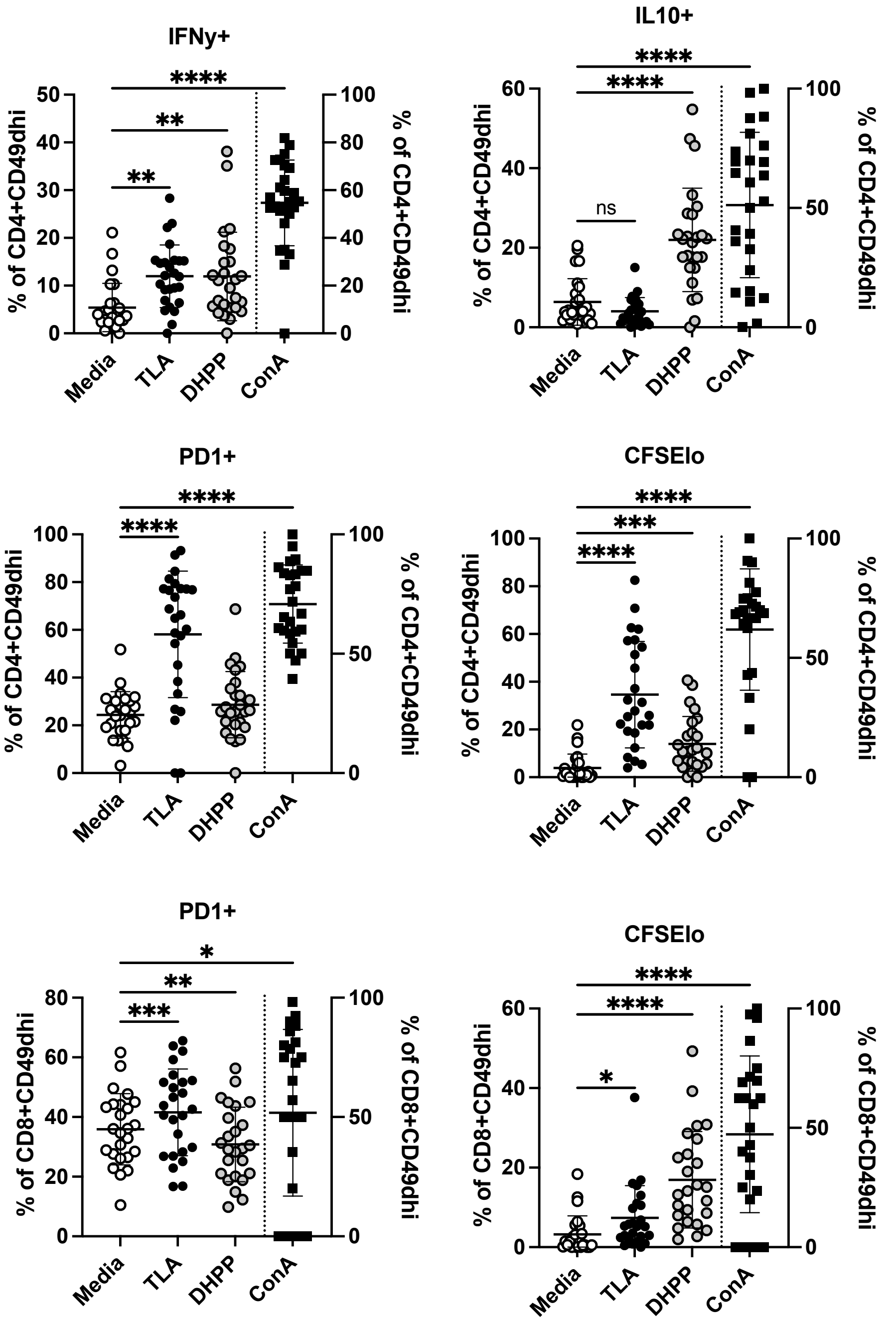


**Supplemental Figure 4. Flow cytometry immune cell readout representative examples.** Media, TLA, DHPP left y-axis. ConA right y-axis. Matched one-way ANOVA with Holm-Sidak post-test.

| | | | | -6 | -3 | TBC | +3 | +6 | +9 | | | |
|---|---|---|---|---|---|---|---|---|---|---|---|---|
| | | | | | | TP1 | TP2 | TP3 | TP4 | TP5 | TP6 | TP7 |
| | | | | | | TP1 | TP2 | TP3 | TP4 | TP5 | TP6 | TP7 |
| | | | | | | TP1 | TP2 | TP3 | TP4 | TP5 | TP6 | TP7 |
| | | | | | | TP1 | TP2 | TP3 | TP4 | TP5 | TP6 | TP7 |
| | | | | | TP1 | TP2 | TP3 | TP4 | TP5 | TP6 | TP7 | |
| | | | | | TP1 | TP2 | TP3 | TP4 | TP5 | TP6 | TP7 | |
| | | | | | TP1 | TP2 | TP3 | TP4 | TP5 | TP6 | TP7 | |
| | | | | | TP1 | TP2 | TP3 | TP4 | TP5 | TP6 | TP7 | |
| | | | | | TP1 | TP2 | TP3 | TP4 | TP5 | TP6 | TP7 | |
| | | | | | TP1 | TP2 | TP3 | TP4 | TP5 | TP6 | TP7 | |
| | | | | | TP1 | TP2 | TP3 | TP4 | TP5 | TP6 | TP7 | |
| | | | | TP1 | TP2 | TP3 | TP4 | TP5 | TP6 | TP7 | | |
| | | | | TP1 | TP2 | TP3 | TP4 | TP5 | TP6 | TP7 | | |
| | | | TP1 | TP2 | TP3 | TP4 | TP5 | TP6 | TP7 | | | |
| | | | TP1 | TP2 | TP3 | TP4 | TP5 | TP6 | TP7 | | | |
| | | TP1 | TP2 | TP3 | TP4 | TP5 | TP6 | TP7 | | | | |
| | TP1 | TP2 | TP3 | TP4 | TP5 | TP6 | TP7 | | | | | |
| TP1 | TP2 | TP3 | TP4 | TP5 | TP6 | TP7 | | | | | | |
| TP1 | TP2 | TP3 | TP4 | TP5 | TP6 | TP7 | | | | | | |

**Supplemental Figure 5. Graphic depiction of TBC+ dog timepoint data included in longitudinal Bayesian mixed-model analysis.** Data collected at each major study timepoint was staggered based on first TBC seroconversion timepoint for each TBC+ subject (n=19). Data from two timepoints previous (-6 and -3 months) to seroconversion and 3 timepoints post seroconversion (+3, +6, and +9 months) were utilized in the longitudinal analysis. Data collected outside this time range was not included in the mixed model analysis (grey). Depending on what timepoint the subject seroconverted to TBC+, data may be missing. The Bayesian mixed model compared the trends for each parameter in the study before (green) and after (orange) TBC seroconversion to output a posterior probability of parameter trajectory post-TBC.

| | | Posterior Probability | | Effect trajectory after coinfection |
|---|---|---|---|---|
| Stimuli | Variable | Increase | Decrease | |
| - | Parasite Burden | **0.84** | 0.16 | Moderately increased |
| - | SLA OD Ratio | **1.00** | 0.00 | Strongly Increased |
| - | LeishVet Stage | 0.53 | 0.47 | no effect |
| TLA | IFNy ELISA | **0.73** | 0.27 | Moderately increased |
| ConA | IFNy ELISA | 0.49 | 0.51 | no effect |
| TLA | IL10 ELISA | 0.45 | 0.55 | no effect |
| ConA | IL10 ELISA | **0.79** | 0.21 | Moderately increased |
| TLA | CD4 %IFNy+ ICS | 0.45 | 0.55 | no effect |
| ConA | CD4 %IFNy+ ICS | 0.21 | **0.79** | Moderately decreased |
| TLA | CD4 %IL10+ ICS | 0.23 | **0.77** | Moderately decreased |
| ConA | CD4 %IL10+ ICS | 0.15 | **0.85** | Strongly decreased |
| TLA | CD4 Proliferation | 0.15 | **0.85** | Strongly decreased |
| TLA | CD4 %PD1+ | 0.46 | **0.54** | no effect |
| TLA | CD8 Proliferation | **0.73** | 0.27 | Moderately increased |
| TLA | CD8 %PD1+ | **0.74** | 0.26 | Moderately increased |

**Supplemental Table 2. Alterations in *Leishmania* diagnostics and immune mediator kinetics following tick-borne coinfection in dogs with asymptomatic CanL.**
Posterior probability (PP) of change in behavior direction and magnitude among TBC-positive dogs after TBC seroconversion timepoint. *PP 0.65-0.85, moderate effect. **PP ≥ 0.85, strong effect.

**Spearman Correlation**

| | Burden | SLA OD Ratio | LeishVet Stage | CD4 ICS IFNy | CD4 ICS IL10 | ELISA IFNy | ELISA IL10 | %CFSElo of CD4+CD49dhi | %PD1+ of CD4+CD49dhi | %CFSElo of CD8+CD49dhi | %PD1+ of CD8+CD49dhi |
|---|---|---|---|---|---|---|---|---|---|---|---|
| Burden | 1.00 | | | | | | | | | | |
| SLA OD Ratio | **0.70** | 1.00 | | | | | | | | | |
| LeishVet Stage | **0.44** | **0.45** | 1.00 | | | | | | | | |
| CD4 ICS IFNy | 0.05 | 0.13 | -0.08 | 1.00 | | | | | | | |
| CD4 ICS IL10 | **-0.23** | -0.14 | -0.13 | **0.21** | 1.00 | | | | | | |
| ELISA IFNy | **-0.35** | -0.16 | 0.08 | **-0.23** | **0.30** | 1.00 | | | | | |
| ELISA IL10 | 0.04 | 0.12 | 0.06 | 0.06 | **0.29** | **0.50** | 1.00 | | | | |
| %CFSElo of CD4+CD49dhi | 0.09 | 0.05 | 0.00 | **0.23** | **0.30** | -0.02 | 0.05 | 1.00 | | | |
| %PD1+ of CD4+CD49dhi | **0.25** | **0.24** | 0.09 | 0.20 | 0.11 | -0.11 | -0.02 | **0.73** | 1.00 | | |
| %CFSElo of CD8+CD49dhi | 0.18 | 0.20 | 0.15 | 0.07 | **0.34** | 0.13 | **0.25** | **0.62** | **0.34** | 1.00 | |
| %PD1+ of CD8+CD49dhi | 0.20 | 0.18 | 0.23 | **0.30** | **-0.24** | **-0.40** | **-0.27** | -0.02 | 0.10 | -0.06 | 1.00 |

**P-value**

| | Burden | SLA OD Ratio | LeishVet Stage | CD4 ICS IFNy | CD4 ICS IL10 | ELISA IFNy | ELISA IL10 | %CFSElo of CD4+CD49dhi | %PD1+ of CD4+CD49dhi | %CFSElo of CD8+CD49dhi | %PD1+ of CD8+CD49dhi |
|---|---|---|---|---|---|---|---|---|---|---|---|
| Burden | NA | | | | | | | | | | |
| SLA OD Ratio | **0.000** | NA | | | | | | | | | |
| LeishVet Stage | **0.000** | **0.000** | NA | | | | | | | | |
| CD4 ICS IFNy | 0.635 | 0.221 | 0.506 | NA | | | | | | | |
| CD4 ICS IL10 | **0.033** | 0.205 | 0.260 | **0.052** | NA | | | | | | |
| ELISA IFNy | **0.003** | 0.171 | 0.500 | **0.050** | **0.011** | NA | | | | | |
| ELISA IL10 | 0.745 | 0.313 | 0.598 | 0.633 | **0.014** | **0.000** | NA | | | | |
| %CFSElo of CD4+CD49dhi | 0.401 | 0.681 | 0.993 | **0.033** | **0.005** | 0.884 | 0.656 | NA | | | |
| %PD1+ of CD4+CD49dhi | **0.041** | **0.050** | 0.500 | 0.097 | 0.382 | 0.396 | 0.880 | **0.000** | NA | | |
| %CFSElo of CD8+CD49dhi | 0.094 | 0.068 | 0.211 | 0.548 | **0.002** | 0.284 | **0.032** | **0.000** | **0.004** | NA | |
| %PD1+ of CD8+CD49dhi | 0.095 | 0.138 | 0.083 | **0.011** | **0.043** | **0.001** | **0.033** | 0.865 | 0.396 | 0.603 | NA |

**Supplemental Figure 6. Relationships between diagnostic and immune mediator levels following tick-borne coinfection in dogs with asymptomatic CanL.** Correlation matrix of diagnostic and *Leishmania* antigen-stimulated immune readouts among TBC+ dogs only including seroconversion and post-seroconversion timepoint data. (Top) Spearman correlation coefficient for each combination. Red indicates a positive correlation; blue indicates a negative correlation. (Bottom) p-value of Spearman correlation. Red hue indicates decreasing p-values. Bolded values indicate p≤0.05.
